# Internal and external drivers interact to create highly dynamic mosquito blood-feeding behaviour

**DOI:** 10.1098/rspb.2024.1105

**Published:** 2024-08-28

**Authors:** Grayson A. Tung, Dina M. Fonseca

**Affiliations:** ^1^ Center for Vector Biology, Department of Entomology, Rutgers University, 180 Jones Avenue, New Brunswick, NJ 08901, USA

**Keywords:** behavioural physiology, disease ecology, Anthropocene, anthropophagy

## Abstract

Blood-feeding, which is necessary for most female mosquitoes to reproduce, provides an opportunity for pathogen transmission. Blood-feeding is influenced by external factors such as light, temperature, humidity and intra- and inter-specific interactions. Physiologically, blood-feeding cycles are linked to nutritional conditions and governed by conserved hormonal signalling pathways that prepare mosquito sensory systems to locate and evaluate hosts. Human activities also alter mosquito blood-feeding behaviour through selection pressures such as insecticide usage, habitat and ecosystem alterations, and climate change. Notably, blood-feeding behaviour changes within a mosquito’s lifespan, an underexplored phenomenon from an epidemiological standpoint. A review of the literature indicates that our understanding of mosquito biology and blood-feeding behaviour is predominantly based on studies of a handful of primarily tropical species. This focus likely skews our comprehension of the diversity of critical drivers of blood-feeding behaviour, especially under constraints imposed by harsh conditions. We found evidence of remarkable adaptability in blood-feeding and significant knowledge gaps regarding the determinants of host use. Specifically, epidemiological analyses assume host use is modified by external factors, while neglecting internal physiology. Integrating all significant factors is essential for developing effective models of mosquito-borne disease transmission in a rapidly changing world.

## 1. Introduction

Organisms that acquire, maintain and transmit pathogens among hosts are known as ‘vectors’. Notably, mosquitoes vector the agents of some of the most severe or impactful human illnesses, including malaria, dengue and lymphatic filariasis [1,2]. While mosquito control efforts in the twentieth century, especially following the development of synthetic pesticides, led to the quasi-eradication of mosquito-borne diseases from the developed world and significant declines in the tropics, cases of mosquito-borne disease have increased steadily over the past 30 years [3]. This highlights the need for novel control strategies based on a better understanding of mosquito biology and behaviour.

**Figure 1.**
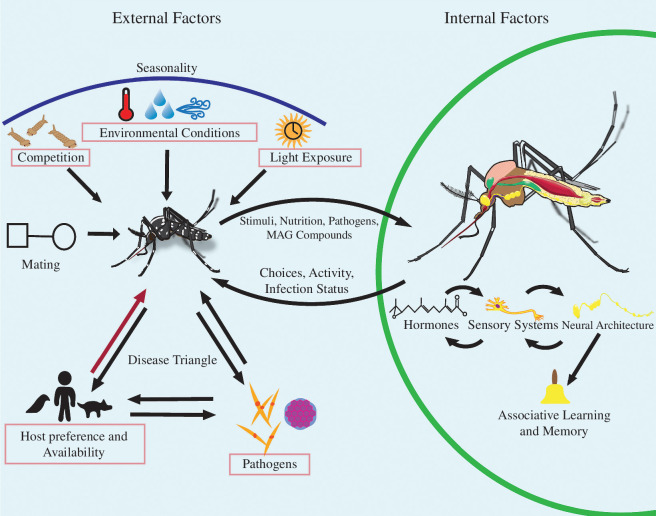
Internal and external factors shape blood-feeding behaviour. On the left side of the figure are external factors, including competition, mating, environmental conditions and light exposure, all influenced by seasonality. These factors alter the female mosquito’s nutritional/physiological status. The disease triangle illustrates interactions among hosts, pathogens and the mosquito (the vector) affecting cycles of transmission. On the right side of the figure, internal factors are illustrated. Hormone production dictates behavioural states during the gonotrophic cycle influencing blood-feeding behaviour. Sensory systems are crucial for host location and assessment. Neural architecture integrates and processes sensory stimuli and shapes associative learning and memory. Internal factors interact to shape observed behaviour. Between external and internal sections, elements like nutrition from blood or sugar, pathogens, male accessory gland (MAG) compounds, and various stimuli affect physiological state and behaviour. Pink boxes highlight categories affected by human activities, including new mosquito species or pathogens, global climate change, pollution, and habitat alteration. Arrows indicate influence direction, with recursive arrows showing reciprocal influences. The red arrow represents blood acquired from hosts.

While some mosquito-borne pathogens can be transmitted from infected females to their eggs, the low rates of vertical transmission of pathogens and high mortality of immature mosquitoes mean that females primarily become infected when feeding on an infected host; transmission to new hosts then occurs during subsequent blood-feeding events [4,5]. It follows that considering the mechanisms that influence blood-feeding is critical to understanding both the maintenance of vector-borne pathogens in the environment and their epidemiology. While being bitten by a mosquito is a familiar experience to many, the factors influencing when, where and why mosquitoes feed on blood are a complex interplay between internal physiological states, external ecological factors and species-specific characteristics (figure 1). A basic understanding of how these factors initiate, operate and interact is needed for the development of more effective mosquito population management.

## 2. Why blood feed?

Finding a host and taking a blood meal is both energetically expensive and potentially dangerous to mosquitoes [6–8]. Despite these costs, a blood meal is often vital for the full maturation of eggs. Notably, insemination is not a mandatory prerequisite for blood-feeding, although most blood-feeding females are mated [9]. This is likely because opportunistic blood-feeding often provides the highest fitness advantage [10], a conclusion we frequently encounter in this review.

Female mosquitoes that require blood to develop eggs are referred to as ‘anautogenous’. In contrast, females capable of producing one or more clutches of eggs without blood are referred to as ‘autogenous’. Autogeny can be fixed or be facultative and is possible when adult females emerge with sufficient nutrition to fully develop eggs. With a few exceptions, autogenous species have predatory larvae or develop in nutritionally dense environments (e.g. water with high organic matter) and are a minority of mosquito species [11]. For anautogenous mosquitoes, blood serves as a crucial source of proteins and lipids to meet the minimum nutrient requirements for oogenesis. Importantly, these nutrients cannot be adequately acquired from carbohydrate-rich food sources like nectar. In addition, several micronutrients and amino acids have a significant role in egg production. For example, fecundity and fitness of offspring are correlated with levels of isoleucine, iron, cholesterol and vitamins in the host blood [12,13].

While a blood meal is primarily used to develop eggs, blood can supplement female nutrition, be incompletely utilized or excreted, or be taken to maintain developed eggs [14–16]. For example, extended starvation increases the likelihood of gravid *Aedes aegypti* taking an additional blood meal [17]. Conversely, sugar feeding may depress blood meal intake, digestion and blood-feeding avidity [18]. Also, when oviposition sites are unavailable, gravid *Anopheles gambiae* may take additional blood meals to retain viable eggs [19,20]. Interestingly, gravid *Culex pipiens* can retain viable eggs for up to eight weeks without an additional blood meal [21].

## 3. Which internal mechanisms govern blood-feeding?

Physiologically, blood-feeding is modulated by numerous internal cues that begin immediately after female ‘eclosion’ and occur cyclically until death. Patterns of gene expression, hormone production and neurological activity vary greatly between pre-blood-meal states, which facilitate the acquisition of blood, and post-blood-meal states, which inhibit host-seeking and blood-feeding in favour of reproductive development. In the most basic sense, blood-feeding meets the nutritional needs of the reproducing female mosquito; proteins and lipids from blood are used to produce yolk proteins. Specifically, ‘vitellogenesis’, the production and transport of vitellogenin (the primary yolk protein precursor of insects) to maturing oocytes, is activated by the acquisition of blood [22].

### (a) Hormonal signals

**Figure 2.**
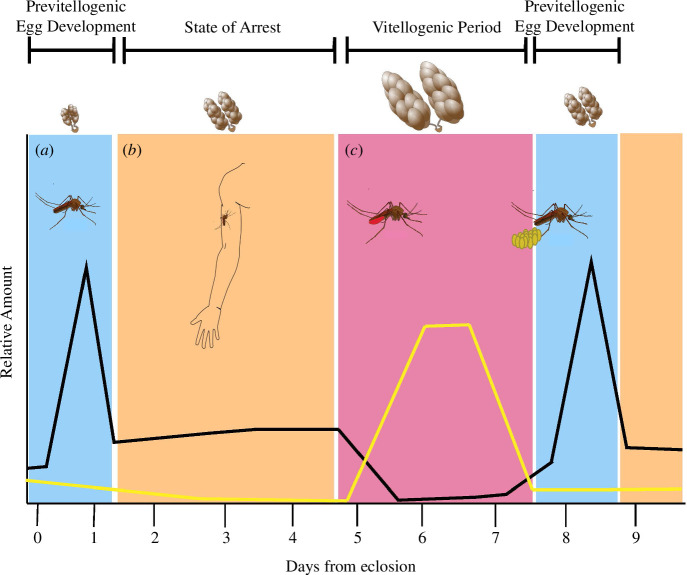
Cycles of hormone production in relation to blood-feeding behaviour and ovarian development. Juvenile hormone titres are represented by the black line, while ecdysone is represented by the yellow line. (*a*) period of unresponsiveness toward hosts as previtellogenic egg development occurs; (*b*) period during which female mosquitoes actively seek hosts and blood feed, ovaries cease development until blood is taken, and JH is produced at constant levels; (*c*) after blood is taken, JH production ceases and ecdysone production begins to stimulate vitellogenesis. After eggs are developed and laid, another cycle of JH production and host-seeking begins.

Endocrine signalling in mosquitoes is critical to the initiation and termination of blood-feeding [23,24]. ‘Juvenile hormone’ (JH) is conserved across all insects and regulates development as insects mature. In adult female mosquitoes, however, JH regulates blood-feeding and reproduction. In newly eclosed adults JH is produced in high amounts and is associated with early egg development [23,25]. During this period (figure 2*a*), JH stimulates the production of yolk protein precursors in the fat body that are secreted into the haemolymph and absorbed by developing oocytes [23]. As early egg development continues, female mosquitoes are far less likely to bite as blood would not be efficiently used in oogenesis [26]. Following previtellogenic egg development, JH titres are maintained at consistent levels (figure 2*b*). This period is associated with active host-seeking and blood-feeding, and if mosquitoes cannot produce JH, they become unresponsive to hosts [24]. Significantly, several odourant binding proteins specifically interact with JH in mosquitoes, suggesting that some olfactory responses may be regulated by JH [27,28]. Furthermore, JH interacts with other signalling pathways that regulate egg development both before and after blood-feeding. Nutrient signalling pathways such as the insulin-target of rapamycin (TOR) pathway have been shown to connect the nutritional state of both larvae and adult mosquitoes with JH production [23,29,30]. Similarly, the forkhead box transcription factor (FOXO), a component of the insulin signalling pathway, has been shown to regulate vitellogenesis, diapause and JH synthesis [31–33].

Following the acquisition of a blood meal, mosquitoes enter another period of unresponsiveness to hosts as blood is used for egg development (figure 2*c*). This period is associated with a decrease in titres of JH and an increase in another hormone, ‘ecdysone’, which regulates vitellogenesis. Once eggs have been completely developed and deposited, JH production resumes and the cycle of host-seeking and biting begins once again.

### (b) Sensory systems

The molecular mechanisms underlying when and which hosts mosquitoes target have been extensively researched in recent years. Visual and chemical cues attract mosquitoes from far away, while physical cues such as temperature are detected near the host. Mosquito species often differ in host use, responsiveness to host cues, and times of day or year when they actively seek hosts [34]. Accompanying these differences are physiological changes in odourant receptors, gene expression and circadian rhythms that work in tandem to increase the likelihood of finding a host [35–37]. Much research has focused on the olfactory receptors of anthropophilic mosquitoes, specifically to identify compounds that activate these receptors, and how these receptors have evolved [38,39]. The primary olfactory organs of mosquitoes are the antennae, although olfactory receptors have been located on maxillary palps and proboscis [40]. Mosquito olfactory receptors respond selectively to different odourants, from general host cues such as CO_2_ to odourants that are more specific to certain taxa, such as sulcatone, a component of human odour [41,42]. However, mosquito attraction to hosts is unlikely to be determined by an individual compound, but rather by blends of these chemicals produced in differing amounts [43,44].

Near the host, mosquitoes integrate both visual and gustatory cues as they approach and land [45]. Once near the host, mosquitoes use ‘ionotropic receptors’ in their antennae to detect heat and humidity cues [46] and ‘gustatory receptors’ on their tarsi and mouthparts [47]. Mosquitoes can also detect sound through the Johnston’s organs. For example, species of mosquitoes that specialize on frogs, namely *Uranotaenia* spp., have been shown to respond to the calls of frogs in both laboratory and field environments [48]. However, the degree to which mosquitoes use sound to detect hosts is poorly understood. In summary, the integration of multiple host cues allows the successful execution of blood-feeding in mosquitoes and the absence of some cues often does not prevent success, highlighting a high degree of redundancy in their sensory mechanisms [49].

### (c) Neural architecture

Recent research has also examined how neural architecture is involved in host-seeking behaviours. Once odourants are detected, signals from the corresponding olfactory sensilla are transduced to the antennal lobe, the primary centre of olfactory information in the insect brain [40]. Within the antennal lobe, axon terminals converge into clusters of nerve endings called ‘glomeruli’. These convergences are associated with the identification of specific compounds or groups of compounds, and sensory neurons expressing similar receptors terminate in specific glomeruli, indicating that certain brain regions are coded to respond to related stimuli such as host odours [50]. This has been recently examined in mosquitoes, where patterns of activation of specific glomeruli correspond with host odourants, and activation of multiple glomeruli may allow mosquitoes to discriminate among hosts [51]. Context-specific patterns of activation have also been observed in mosquitoes exposed to plant odours, suggesting that patterns of glomerular activity are also associated with differentiation between nectar sources [52]. These studies have begun to decode the mechanisms behind the ability of mosquitoes to integrate olfactory information from multiple hosts to inform behaviour. However, our understanding of the more complex aspects of odour processing in different regions of the brain remains limited. For example, the mushroom bodies and lateral horns are regions of the insect brain that receive signals from the antennal lobes and are associated with learning and memory in other insects [53]. While we know mosquitoes are capable of ‘associative learning and memory’, e.g., females are more likely to feed on species they have fed on successfully before [54] and will actively reject an odour after a negative experience [55], the specific mechanisms underlying these processes in the brain remain poorly understood.

## 4. How does the external environment affect blood-feeding?

Several physical external factors influence the ability of mosquitoes to find and exploit hosts as a source of blood. While some aspects of the environment affect blood-feeding by creating suitable conditions for host location and blood acquisition, in many cases mosquitoes cease blood-feeding to facilitate survival in poor conditions [56,57]. Adaptations to specific environments have been shown to have a genetic basis, and conspecific populations may exhibit marked differences in behaviour based on the physical characteristics of their environment [58–60].

### (a) Environmental conditions

Mosquitoes, like all insects, are ectotherms, meaning they depend primarily on their environment for temperature regulation. Consequently, environmental temperature significantly influences their physiology, behaviour and ecology, including blood-feeding. Mosquitoes have developed various mechanisms to adapt to temperature fluctuations. Species often have an optimal temperature range for specific activities, and their ability to function is restricted beyond certain lower and higher temperature thresholds [61]. Furthermore, the speed and wingbeat frequency of mosquito flight, necessary to acquire a host, can be affected by temperature [62]. Temperature has an additional impact on blood-feeding females as it influences the ability to detect hosts using cues generated by temperature differentials between the host and their surroundings [63]. Notably, thermoreceptors in the antennae of *Ae. aegypti*, a tropical day-biting species, exhibit exceptional sensitivity, capable of detecting temperature differences as small as ±0.2°C [64]. Furthermore, to safeguard against heat shock associated with the ingestion of a warm blood meal, several mosquito species, including *Ae. aegypti* and *Cx. pipiens*, produce heat shock proteins [65,66]. Additionally, *Culex quinquefasciatus* has been shown to employ evaporative cooling through the generation of fluid droplets to mitigate excess heat [67].

While the effect of temperature on adult mosquito survival has been extensively studied, the impact of humidity is not as thoroughly understood. It is important to note that temperature and humidity are interrelated: warmer air requires higher moisture levels to achieve saturation and therefore has a greater drying effect relative to cold air. While the tolerance for water loss has been thoroughly investigated in various arthropods, few studies have focused on adult mosquitoes [68–70], even though mosquitoes face significant challenges in maintaining water balance [71]. In general, most insects can withstand 20–30% dehydration [69] and *Anopheles. arabiensis* and *An. gambiae,* two important human malaria vectors in sub-Saharan Africa, can endure water losses of 29 and 33%, respectively, before succumbing to dehydration [72]. Interestingly, adult *Cx. pipiens* can withstand higher water loss, up to 40% of their body mass, without fatal consequences. Remarkably, mosquitoes do not replenish water reserves through metabolic water production or atmospheric vapour absorption. Instead, they rely on the ingestion of liquid water or blood for rehydration [68]. Therefore, dehydrated female *Cx. pipiens* actively seek blood hosts and display increased host-landing and blood-feeding behaviours, which may impact pathogen transmission [73].

Wind speed can also interfere with mosquito blood-feeding although the precise mechanism remains unclear. When confronted with wind exceeding 1.5–2.8 m s^−1^, mosquitoes tend to either land or fly at lower altitudes, where wind speeds are slower [74]. This behaviour has been proposed as an explanation for the observed inverse relationship between host-finding success (or its proxy, mosquito trap catches) and wind velocity [75]. However, wind can also dilute host-associated odours, which can inhibit host-seeking behaviour [76].

### (b) Time of day

Closely associated with temperature is diel activity, the period in which mosquitoes are actively host-seeking. Traditionally, mosquitoes have been broadly categorized as ‘day-biting’ or ‘night-biting’. This is normally determined by actively collecting mosquitoes at different times throughout the day/night or using host-seeking mosquito traps. However, classifying mosquitoes as day- or night-biting can be misleading, as periods of activity are not equal across the entire day or night, and mosquitoes typically have a window of time when they are most likely to blood feed [77,78]. Laboratory experiments altering the exposure of mosquitoes to varying durations of light and dark have shown that biting behaviour is closely tied to photoperiod, and host-seeking behaviours can be altered by light exposure [79–81]. Similarly, mosquitoes enter a daily sleep-like state during their inactive periods, and interruptions of this period of inactivity influence host landings and propensity to blood feed [82]. However, experiments have shown that mosquitoes are entrainable to differing photoperiods, which means they can adjust or synchronize their activity to different lengths of day and night, or variations in the amount of light they are exposed to during a 24 h period, suggesting that species can adapt to differing light exposure depending on their environment (i.e. artificial light, changes in latitude mediated by human activity) [83].

### (c) Seasonality

For mosquitoes in temperate zones, ‘diapause’, a period of reduced activity and metabolism [84], allows individuals to survive periods of extended cold, unavailability of food resources, or limited oviposition sites. Diapause in mosquitoes has evolved independently several times, and the mechanisms governing this strategy vary across groups [84]. Diapause has allowed mosquitoes to expand into higher latitudes and synchronize with suitable climatic conditions and host availability. Adult mosquitoes that enter diapause to survive the winter, trade blood- feeding and focus on sugar consumption to reserve lipids necessary for surviving this unfavourable period [57]. For example, *Cx. pipiens*, the northern house mosquito, enters diapause once daylength falls below a critical threshold that at some latitudes occurs in late summer [57]. Decreasing photoperiods are detected by late fourth instar larvae and early pupae and result in lower production of JH in newly emerged adults, which in turn decreases host-seeking [85]. Newly emerged females during this time will feed on sugar, mate and ultimately overwinter as adults. This is a particularly interesting shift, because while this behaviour allows a female mosquito to survive the winter, it reflects an expectation of future environmental conditions that reduces the number of gonadotropic cycles that in some years might be completed prior to the onset of unfavourable conditions.

In arid environments, mosquitoes must adapt their behaviour to survive extended dry periods. Anopheline mosquitoes in the African Sahel, for instance, employ two strategies to cope with prolonged low precipitation: migration or ‘aestivation’, which is an extended dry season dormancy [86]. Laboratory experiments have also revealed that, during the dry season, female mosquitoes significantly reduce their likelihood of taking a blood meal [56]. This behavioural change offers dual benefits. Firstly, it minimizes their exposure to desiccating conditions while foraging for blood hosts. Secondly, it aligns with the scarcity of oviposition sites during this period. A similar adjustment in feeding behaviour is observed during the dry summer in southern California, when *Culiseta inornata* mosquitoes become less inclined to take blood meals. Instead, they prioritize sugar-feeding and accumulating lipid reserves, critical for their survival during the extended dry period [87].

Even during their active stage, numerous mosquito species exhibit variation in host preference and biting patterns in response to seasonal changes. Ornithophilic species such as *Cx. pipiens, Culex nigripalpus* and *Culex tarsalis* have been observed to shift their feeding behaviour towards mammals, including humans, as autumn approaches [88–90]. These alterations in feeding patterns hold significant epidemiological implications, as they appear, for example, to contribute to late-season spikes in human cases of West Nile virus [88]. Although the cause of these behavioural changes remains uncertain, researchers have posited that they may be influenced by factors such as host availability [90] or moisture levels that influence flight [91] but have so far failed to consider internal factors such as physiological responses to decreasing daylight or increasing mosquito age in the autumn.

## 5. How do inter- and intra-specific interactions influence blood-feeding?

Mosquitoes, like all organisms, exist in networks of individuals and species that can shape their behaviours. At its core, blood-feeding is a highly specialized inter-specific interaction, where adult female mosquitoes are dependent on the presence and abundance of suitable hosts. Additionally, blood-feeding is inextricably tied to nutrient acquisition and reproduction and therefore intra-specific interactions such as mating and competition. As vectors, mosquitoes are also exposed to pathogens which may affect their survivorship or alter their behaviour to facilitate transmission.

### (a) Mating

Female blood-feeding can be influenced by seminal fluids, hormones and proteins that are transferred during mating; many of these compounds are produced in the male accessory glands (MAGs). Mating or injection of MAG extracts into female *Ae. aegypti* was shown to stimulate oviposition, prevent further mating, and lessened host-seeking behaviour [92,93]. After mating or after being injected with MAG extracts, female *Cx. quinquefasciatus* shifted flight activity to earlier in the evening [94]. In *Aedes taeniorhynchus*, MAG extracts are essential for autogenous egg development [95,96], pre-empting the need for blood-feeding. Indeed, while some compounds transferred from the male during mating may not directly affect blood-feeding behaviour, they are often closely associated with gonotrophic development, which in turn modulates cycles of blood-feeding [93,97,98].

### (b) Competition

Conditions associated with larval development have an impact on the success of all adults post-emergence. For example, temperature, larval nutrition, larval density and inter-specific competition affect adult size [99–102]. Adult size can influence ‘vectorial capacity’, a measure of the potential for a vector to contribute to a pathogen’s transmission. Larger mosquitoes have been shown to live longer [103,104], increasing the opportunities for pathogen transmission. Conversely, smaller females have been observed to require more than one blood meal to complete a gonotrophic cycle, which increases the number of opportunities for a female mosquito to acquire and transmit pathogens [105,106], possibly owing to limited JH production [107]. In support of this hypothesis, it was found that treating small-bodied *Ae. aegypti* with JH immediately after their emergence as adults enables them to develop eggs after a single blood meal [29].

While inter- and intra-specific resource competition is a common and well studied phenomenon in mosquito larvae [99,104,108], blood-feeding competition (or interference) between adult mosquitos is not well understood. Soghigian *et al*. [109] found evidence of inhibition of blood-feeding in female *Ae. aegypti* exposed to male *Aedes albopictus*. Because the authors did not assess if satyrization had occurred, i.e. if male *Ae. albopictus* had mated with female *Ae. aegypti*, it is unclear if the effect was due to the transfer of MAG compounds or direct physical interference. Furthermore, the degree to which this occurs in nature is unknown.

### (c) Host preference and availability

How host preference or host availability drives mosquito choices has been the subject of much research. In behavioural assays, mosquitoes are often capable of distinguishing host stimuli and consistently show a preference [39,41,110]. Often species are categorized based on their general feeding preferences (i.e. ornithophilic, mammophilic, anthropophilic, etc.), which determine vectorial capacity. Blood-meal analyses can determine which hosts make up the majority of blood meals and provide species-specific feeding patterns in relation to host abundance [111–113]. While blood-meal analysis is useful for pinpointing important reservoir hosts and assessing vectorial capacity, it can be difficult to accurately count available hosts, which makes statements of preference hard to support.

Furthermore, assessing the fitness costs associated with mosquitoes feeding on different hosts in nature poses considerable challenges. While it might be intuitive to assume that feeding on a preferred host should enhance reproductive success, laboratory studies present a conflicting picture [114–116]. Specifically, the effect of blood source on reproductive output, even as different as birds versus mammals, appears minimal. Of course, laboratory experiments, while informative, commonly use colonized populations and lack the ecological context crucial for understanding mosquito blood-feeding decisions, such as a host cues, abundance or defensive behaviours. Intriguingly, colonized mosquito strains have increased reproduction when fed on blood from the host species used to maintain the colony, which frequently includes species rarely encountered in the wild [117,118].

### (d) Pathogen interactions

The effect of pathogens on mosquito feeding behaviour is an emerging and important research theme. In anopheline mosquitoes, studies have shown that *Plasmodium* infection increases avidity to human hosts and alters the expression of genes involved in host-seeking [119–121]. In *Ae. aegypti*, infection with dengue virus affects gene expression in the salivary glands, increasing avidity and time spent probing compared with non-infected females [122–124]. It remains less clear how infected hosts modulate mosquito behaviour. A recent review concluded that variation in experimental conditions and methodologies poses challenges to determining the true influence of the bird host’s *Plasmodium* infection status on mosquito avidity [125].

## 6. Does human activity affect mosquito blood-feeding?

Humans may modify mosquito behaviour patterns by creating new habitats that mosquitoes can exploit. Evolutionarily, species that have co-evolved alongside humans are not only responsive to human cues but also adapted to survive and reproduce in man-made environments, i.e. are ‘endophilic’ or ‘synanthropic’ [35,126]. Examples of this include *Cx. pipiens* f. *molestus*, which thrives in subterranean structures such as subways and sewers, *Ae. aegypti*, which can develop in indoor drinking water containers [127], peri-domestic *Ae. albopictus*, which develop large populations in small artificial containers [128], and *Cx. quinquefasciatus*, which thrives in urban ditches and pit latrines [129]. Of note, experimental exposure to artificial light sources at night has been shown to cause female *Cx. pipiens* reared under short daylight conditions and that have accumulated fat reserves to still blood-feed and lay eggs [130].

Climate change will likely impact mosquito blood-feeding by altering temperature and humidity, host abundance, and expansion of habitable ranges [131,132]. While photoperiodic diapause is driven by daylength, increased temperatures could change the internal response to this stimulus and increase the length of the active season. As evidenced by invasive species moving across latitudes [133], mosquito populations can adapt to new climates. Therefore, it is important to routinely reconsider observations related to seasonality and temperature, as these behaviours may change.

Mosquito control can also drive changes in behaviour that ultimately affect blood-feeding. For example, insecticide-treated bed nets and residual indoor pesticides have been invaluable tools in reducing the transmission of human malaria in Africa [134]. However, these interventions can be selected for outdoor day-biting [135,136]. Sougoufara *et al*. found that mosquitoes will alter their behaviour to avoid insecticides after a single sub-lethal exposure [55], indicating that human interventions can change behaviours within the lifetime of a single individual.

## 7. How can a better understanding of mosquito blood-feeding be used to improve public health?

The human biting rate of mosquitoes is often treated as a fixed value, historically determined by measuring landing or biting rates of mosquitoes in a field environment [137,138]. The ability of a given mosquito population to transmit a disease agent, i.e. its vectorial capacity (*V*), is calculated using the formula:


$$V=\frac{ma^{2}p^{n}}{-\ln\left(p\right)}.$$

Here, *m* represents the ratio of vectors to humans, *a* is the rate at which the vector bites humans, *p* is the daily survivorship of the vector, and *n* is the extrinsic incubation period of the pathogen [139]. Of note, the use of the ‘extrinsic incubation period’ adds a measurable, predictable parameter to the classical Ross–Macdonald equation, which instead used ‘parasite transmission probability’ (*b*).

However, these representations of vectorial capacity exclude zoonoses, which involve non-human hosts, and variation in host use over time and space influenced by the factors described previously [73,90,108]. One example of integration of host use into the calculation of mosquito-borne disease risk is a formula that includes a fractional representation of human blood meals to estimate infection prevalence and transmission of bird arboviruses, such as West Nile virus, to humans [140].


$$Risk=A\times F_{M}\times P\times C_{v}$$

Here, *A* represents the abundance of a mosquito species, *F*_M_ is the proportion of blood meals taken from mammals, *P* is the prevalence of infection and *C*_v_ is vector competence. This method estimates risk by recognizing that female mosquitoes that have fed on non-human hosts have a higher likelihood of carrying zoonotic pathogens. When these mosquitoes are present in significant numbers near human populations, even seemingly minor instances of mosquito bites on humans can have significant epidemiological implications.

Critically, even complex individual-based models that simulate the behaviour and ecology of adult mosquitoes in exquisite detail on complex resource landscapes fail to consider physiological factors [141]. For example, low nutritional status due to larval competition can result in an additional blood meal per gonotrophic cycle, increasing transmission. In this case, incorporating data on average adult size or larval density into the model would enhance its usefulness as an epidemiological tool. Furthermore, existing models overlook the epidemiological significance of measurable sub-populations of females that are unlikely to blood-feed at one time, increasing their potential for effective pathogen transmission when they do.

## 8. What do we know now, and where do we go from here?

From the previous sections, it is evident that constructing a comprehensive theory to explain the intricacies of how, what, when and why mosquitoes engage in blood-feeding is a complex task, marked by variability across different species, populations and even experiences over a mosquito’s lifetime. Significantly, existing paradigms in mosquito blood-feeding research tend to overlook these variations.

As blood-feeding is a behaviour restricted to female mosquitoes, we have a better understanding of the biology of female mosquitoes in relation to blood-feeding than that of males. However, as summarized, males often influence female behaviour. For example, male *Ae. aegypti* and *Ae. albopictus* find and mate with females near hosts [142]. This indicates that males are also able to recognize and locate hosts and may be drivers in the development of host specificity. In this way, both male and female mosquitoes can actively contribute to the process of host preference and specialization.

Regarding host use, because blood-feeding is energetically expensive and dangerous, it would make sense for females to feed opportunistically and not expend time and resources locating specific hosts when more readily available options exist. Nevertheless, most mosquitoes display some degree of host preference. There are many questions about the advantages of specializing in a narrow range of hosts compared with adopting more generalized feeding behaviours. Studies are needed to reconcile the benefits of host specificity with the costs of delayed feeding considering the idiosyncrasies of evolution (i.e. behaviours that were once adaptive may not be currently). Importantly, while most mosquitoes do not focus on humans, our understanding of enzootic vector cycles remains relatively limited. Given that many mosquito species primarily feed on non-human animals, significant research is required to discern which species play a role in pathogen transmission among reservoirs and which may be responsible for zoonotic spillover to humans.

Considering the existence of over 3500 mosquito species [143], most of our knowledge about mosquitoes is derived from experiments and observations conducted primarily on a handful of species (electronic supplementary material, S1). These few species have become the *de facto* standards for studying mosquito physiology and behaviour because of their significance as vectors, ease of colonization and strong use of humans as blood host habits. This focus likely restricts and biases our understanding of blood-feeding behaviour, especially considering these species are predominantly tropical. Because environmental factors influence blood-feeding, the overrepresentation of tropical species in mosquito biology neglects the adaptations of mosquitos to extended periods of unfavourable conditions in temperate climates. Consequently, a wide array of behavioural, physiological and ecological capabilities remain largely unknown. This is especially relevant as the world is changing.

Nonetheless, the information we do have suggests there are conserved features of blood-feeding that are important across mosquitoes. In general, the initiation and suppression of the biting cycle of mosquitoes is regulated internally, with nutritional and hormonal signalling pathways playing an important role. Specifically, the inverse relationship between JH and ecdysone in the regulation of biting cycles and oogenesis has been observed in multiple species [24,29]. The evolutionary cooption of these two hormones, traditionally associated with the development of immature insects, is common across insect taxa and often tied to reproduction in adults. However, little is known about how JH affects behaviour either directly or indirectly through JH-responsive elements. Studies are needed to identify genes that are transcribed in response to JH-associated promotors and if JH alone is sufficient to trigger host-seeking and blood-feeding.

## Data Availability

The list of publications assembled from the Web of Science using the search terms ‘mosquito’ and ‘blood feeding’ from 1965 to the present is available from the authors upon request. Supplementary material is available online [144].
